# Impact of reflex locomotion and the Bobath concept on clinical and biomolecular parameters in people with multiple sclerosis: study protocol for a randomized controlled trial

**DOI:** 10.3389/fneur.2023.1209477

**Published:** 2023-08-04

**Authors:** Aymara Abreu-Corrales, Ana Velasco, Alicia Cuesta-Gómez, Juan Luis Sánchez-González

**Affiliations:** ^1^Programa de Doctorado Neurociencias, University of Salamanca, Salamanca, Spain; ^2^Department of Biochemistry and Molecular Biology, Institute of Neurosciences of Castilla and Leon (INCYL), Institute of Biomedical Research of Salamanca (IBSAL), University of Salamanca, Salamanca, Spain; ^3^Motion Analysis, Ergonomics, Biomechanics and Motor Control Laboratory (LAMBECOM), Department of Physical Therapy, Occupational Therapy, Rehabilitation and Physical Medicine, Faculty of Health Sciences, Rey Juan Carlos University, Madrid, Spain; ^4^Department of Nursing and Physiotherapy, University of Salamanca, Salamanca, Spain

**Keywords:** physiotherapy, reflex locomotion, Bobath concept, multiple sclerosis, neurorehabilitation

## Abstract

**Introduction:**

Multiple sclerosis (MS) is a progressive disease with a fluctuating and unpredictable course that has no curative treatment at present. One of its main characteristics is the variety of signs and symptoms that produce a high percentage of patients who present alterations in balance and gait during the development of the disease, decreased muscle strength, spasticity, or decreased pimax. Rehabilitative therapy, especially physiotherapy, is the main course of the treatment of these alterations using reflex locomotion and the Bobath concept as a form of kinesitherapy that activates the preorganized circuits of the central nervous system.

**Objective:**

The objective of this study is to evaluate the reflex locomotion and Bobath concept effects on balance, spasticity, reaction time, respiratory parameters, and lacrimal biomolecular markers.

**Methods and analysis:**

This is a randomized controlled trial on the effectiveness of two neurorehabilitation techniques in patients with multiple sclerosis conducted at the University of Salamanca. The research will take place at the Faculty of Nursing and Physiotherapy, University of Salamanca. The study will be conducted from June 2023 to June 2024. The reflex locomotion group will receive individual sessions of therapy (*n* = 27), and the Bobath concept group (*n* = 27) will receive the same number of sessions. Both groups will receive two sessions per week for 12 months. The measurement variables will be the Berg Balance Scale, the Tardieu Scale, the Cognitfit Program, Maximum Inspiratory Pressure, and Lacrimal Biomarkers.

**Ethics and dissemination:**

This study has been approved by the Ethics Committee of the University of Salamanca on March 2023 (ref: 896).

**Limitations:**

The main limitations of this study are the selection and number of patients, the delay in implementing the therapy within the initially scheduled period, inadequate sample collection, and inadequate sample processing.

**Trial registration number:**

ClinicalTrials.gov; identifier: NCT05558683.

## Highlights

- Examining the effectiveness of two physical therapy treatments in patients with multiple sclerosis through a randomized controlled trial could provide insight into which types of techniques are most effective for these patients.
- Primary outcome variables will be measured objectively through validated and adapted tests/instruments, providing a valid basis for data interpretation.
- Biochemical parameters will be measured at the Institute of Neurosciences of Castilla y León by qualified personnel trained in this type of measurement and not by the study team.

## Introduction

Multiple sclerosis (MS) is considered a chronic, multifocal demyelinating disease of the central nervous system (CNS). Its diagnosis is fundamentally clinical. The level of disability is rated using the Expanded Disability Status Scale (EDSS), a scale useful for measuring neurological disability (cerebral, cerebellar, pyramidal, brainstem, sensory, bowel, visual, and bladder functions) (1).

Given that sometimes drug treatment does not have the desired efficacy, some authors highlight the importance of physiotherapy (2) as an important part of the symptomatic and supportive treatment of people with MS since it induces improvement of the affected physiological functions occurred due to lack of physical activity and helps control some symptoms, such as spasticity, fatigue, and lack of balance (3).

The reflex locomotion (Vojta Method) and the Bobath concept are considered two variants of special interest in their approach (4–6).

The reflex locomotion created by Dr. Vojta between 1950 and 1970 is based on the principle of reflex locomotion, so it is possible to reflexively trigger repeated motor reactions in the trunk and extremities from defined stimuli and from certain postures. The movement complexes that are activated are reflex crawling and reflex turning, producing numerous effects and benefits at a global level (7, 8).

The neurophysiological mechanisms of this reflex locomotion have recently been discovered. In their study, Sanz-Esteban et al. (9) concluded that the application of the therapy activates the basal ganglia, the putamen, the anterior cerebellum, and the thalamus. These structures play an essential role in motor actions. In a recent study (10), stimulation of the chest area was used in the oscillation reflex coordination complex, showing activation of cortical areas such as supplementary motor areas (SMA) and premotor areas (PMA; Brodmann areas BA6 and BA8), which are areas in charge of movement planning, regulation, and execution, as demonstrated by Martínek et al. (11).

In addition, it has been shown that reflex locomotion produces an involuntary contraction of the abdominal musculature measured by electromyography (12). In other studies by Laufens et al. (13, 14), we can verify the great benefits obtained by combining reflex locomotion with Laufens electromyographic results in the improvement of fatigue and muscle strength, reporting improvements of up to 68.4% in cerebellar functions and 78% in lower limb strength.

However, the Bobath concept is a valid and recognized option in the treatment of patients with neurological disorders and, therefore, of those diagnosed with MS. It was developed by Karel and Berta Bobath. The Bobath concept is defined as a problem-solving approach in the evaluation and treatment of people with altered neuromotor function, becoming a valid tool as part of the comprehensive treatment of patients with MS (15).

Castelli et al. show the positive impact of the application of the Bobath concept on balance, postural control and indirect adaptive neuroplasticity index in people with multiple sclerosis (6).

It is important to mention that there are other relevant studies with butolinic toxin treatment combined with reflex locomotion and the Bobath concept to improve spasticity in patients with multiple sclerosis (16).

Through this concept, active movements are carried out, which requires the participation of the patient, where the individual fulfills the motor, sensitive, and cognitive functions; the executed tasks lead to stability and mobility, incorporating knowledge of motor learning, biomechanics, motor control, and brain plasticity depending on the activity that requires recovery after brain injury (17).

Also relevant is the appearance of abnormal postural and movement patterns due to the release of abnormal postural reflex activity (18).

We must highlight that reflex locomotion and the Bobath concept are shown to be one of the most widely used therapies in the rehabilitation process of potentially altered variables such as spasticity, reaction time, muscle strength, balance, and pimax during the course of diseases such as multiple sclerosis. However, to date, there has been no study that has evaluated the effectiveness of both therapies in a population diagnosed with multiple sclerosis. In addition, most studies demonstrate the clinical improvement experienced by people with multiple sclerosis; however, the reason for this clinical improvement is not explained. Therefore, studies like ours could be a therapeutic tool that would facilitate the direct activation of innate patterns in the central nervous system. This would allow us to activate partial movement patterns and use them to achieve better postural control, stabilization, improved support, and coordinated phasic movement. Although the relationship between the application of reflex locomotion and the activation of central nervous system structures during the development of multiple sclerosis has been demonstrated, it is interesting to consider its combined use with other therapies.

One of the tools used as part of the exploratory analysis in clinical practice that refers to the state of health of the disease and its evolution is the multiple sclerosis response biomarkers. In this research project, we will analyze biomarkers in tear fluid, which would allow the progression of the disease to be quickly and inexpensively deciphered. Tear fluid constitutes a biological sample related to the nervous system and is easily obtained, constituting a very interesting source of study for the detection of biomarkers of neurodegeneration (19–21).

There are few comparative studies on the effectiveness of reflex locomotion vs. the Bobath concept as rehabilitative therapies in people with MS, and none to date have analyzed in parallel the expression of tear biomolecular markers that could participate in the myelination process, balance, respiratory parameters, spasticity, and reaction time. Hence, we highlight the relevance of our hypothesis while proposing a new methodology in physiotherapy (22, 23).

## Hypotheses

Reflex locomotion and the Bobath concept are two techniques used in rehabilitation for the evaluation and treatment of people with disorders of function, movement, and postural control as a result of a lesion in the central nervous system. To date, there are few comparative studies on the effectiveness of both techniques as rehabilitation therapies for people with multiple sclerosis. None of these studies have analyzed whether the application of these therapies entails a modification of the levels of certain tear substances indicative of processes of myelination, balance, respiratory parameters, spasticity, and reaction time that could act as biomarkers of disease progression.

Our hypothesis is that the application of reflex locomotion in subjects with MS causes a greater improvement in their physical condition and, in general, in their quality of life compared to the Bobath method. This improvement is justified because the first (reflex locomotion) modifies the activity of the central nervous system (CNS), while the second (Bobath concept) acts only at the muscular level. Likewise, we propose that the improvement can be monitored by an alteration in the levels of certain compounds extracted from the tears of the subjects, such as sphingomyelin, which have previously been described as tear biomarkers in the etiology of MS (24).

## Methods and analysis

### Study design and setting

A double-blind randomized controlled trial will be conducted at the Faculty of Nursing and Physiotherapy, University of Salamanca (Spain), according to the consolidated standards of reporting trials (CONSORT) statement (25). The current treatment protocol is described according to the recommendations of SPIRIT (26).

The protocol of this trial received the approval from the Ethics Committee of the University of Salamanca (record number 896) and will be conducted according to the Declaration of Helsinki. The clinical trial was registered at ClinicalTrials.gov (registration number NCT05558683).

The study will be conducted between 1 July 2023 and 1 June 2024 in the Faculty of Nursing and Physiotherapy at the University of Salamanca.

### Participants: inclusion/exclusion criteria

A total of 50 participants will be recruited from the general population with a diagnosis of multiple sclerosis according to the Expanded Disability Status Scale who attend their neurology consultation at the University Hospital of Salamanca. The participants in this study will be adults, aged between 20 and 60 years, of both sexes. All subjects will sign a written informed consent form before any data are collected.

Exclusion criteria will be as follows: (1) Participants with multiple sclerosis with an EDSS scale score equal to or higher than 7.0 will be excluded, (2) Pregnant women, (3) Oncology patients, (4) Non-acceptance and non-signing of the informed consent, (5) Refusal to participate in the study, (6) Patients with severe cognitive impairment that does not allow them to understand the development of the study, (7) Patients with cardiovascular instability, (8) Patients who do not complete at least 75% of the scheduled sessions, (9) Patients who do not present any of the contraindications contemplated for reflex locomotion.

### Interventions

This randomized clinical trial will have two groups, namely, the reflex locomotion group and the Bobath concept group.

The therapies will be described following the recommendations of TIDieR (27) (template for intervention description and replication checklist).

#### Reflex locomotion group

**Brief name:** Reflex Locomotion.

**Why:** Reflex locomotion contains two patterns of global movements, namely, reflex crawling, which is triggered from the prone position, and the turning reflex, which is triggered from the lying down supine and lateral positions. These two patterns are called coordination complexes. Both are innately present in the functioning of the CNS.

Activation of coordination complex muscles that occur in reflex locomotion is generated by touch and proprioception elicited by the hands of the therapist in some specific areas or activation points, and some vector directions are established. Trigger points can be stimulated individually or in combination; by doing so, the latter form would generate a greater input to the CNS and would increase the benefits for the activation of gait patterns. “The kinesiological character on Reflex Locomotion contains essential elements in locomotion, among those that include postural adjustment, straightening on a point of support and the coordinated phasic movement.”

**What (materials):** The only material necessary for the application of reflex locomotion will be a stretcher or floor mats on which the patient will be supported while the physiotherapist applies the intervention.

**Figure 1:**
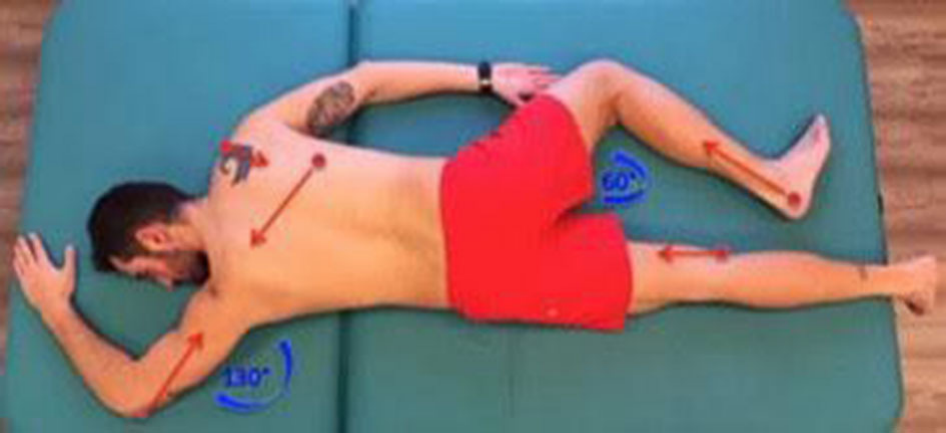
Neurorehabilitation treatment according to Vojta's reflex locomotion.

**What (procedures):** For the application of reflex locomotion therapy, stimulation will be used in the chest area following the pattern of the flip reflex locomotion complex (RV) in its first phase. To do this, the subject will be placed in a supine position aligned with respect to the axial axis, with the arms along the body, the lower extremities extended, and the head extended with a rotation of ~30° to one side of the stimulation. A stimulation pressure between 1.4 and 1.8 kg/cm^2^ ± 200 g will be exerted in the space between the 6th and 7th or 7th and 8th ribs below the mammillary line. Individuals will also be placed in the reflex crawling (RR) position with the patient in the prone position and the head turned 30°. They will be stimulated in the calcaneus area with the same pressure as in the first phase of the reflex rollover. Figure 1 shows the treatment according to Vojta's reflex locomotion. In our study, the starting position of the patient can be modified depending on the degree of mobility and spasticity that the patient may present.

**Who will provide:** All interventions will be performed by a physiotherapist with more than 20 years of experience in treating people with neurological problems.

**How:** The physiotherapy technique will be performed individually twice a week for a duration of 45 min for a period of 12 months (22, 23).

**Where:** All sessions will be conducted at the Faculty of Nursing and Physiotherapy, University of Salamanca (Spain).

**When and for how long:** Participants will receive two sessions per week for 12 months. The average duration of the session will be ~45 min. The same weekly schedule will always be respected for each of the participating subjects.

**Tailoring**: Due to the nature of the intervention and the wide heterogeneity of the subjects, the sessions will be individualized and tailored to each subject.

**How well (planned):** Therapy supervision will be provided through weekly meetings between therapists and investigators. In addition, a thorough control of the subjects' attendance at the sessions will be carried out.

**How well (actual):** Not applicable.

#### Bobath concept group

**Brief name:** Bobath Concept.

**Why:** The Bobath concept is a therapeutic approach used in the rehabilitation of people with neuromotor disorders based on brain plasticity, normal motor control, typical motor development, biomechanical analysis, facilitation and inhibition, and a holistic approach to neuromotor disorders. Its main goal is to improve motor function and promote the person's active participation in his or her environment.

**What (materials):** To apply Bobath therapy, a variety of materials and equipment are required to help the therapist facilitate and promote motor function in people with neuromotor disorders. These include mats, therapeutic balls, mirrors, parallel bars, balance cushions, elastic bands, and pulleys. It is important to note that the materials and equipment used may vary depending on the specific needs of each patient.

**Figure 2:**
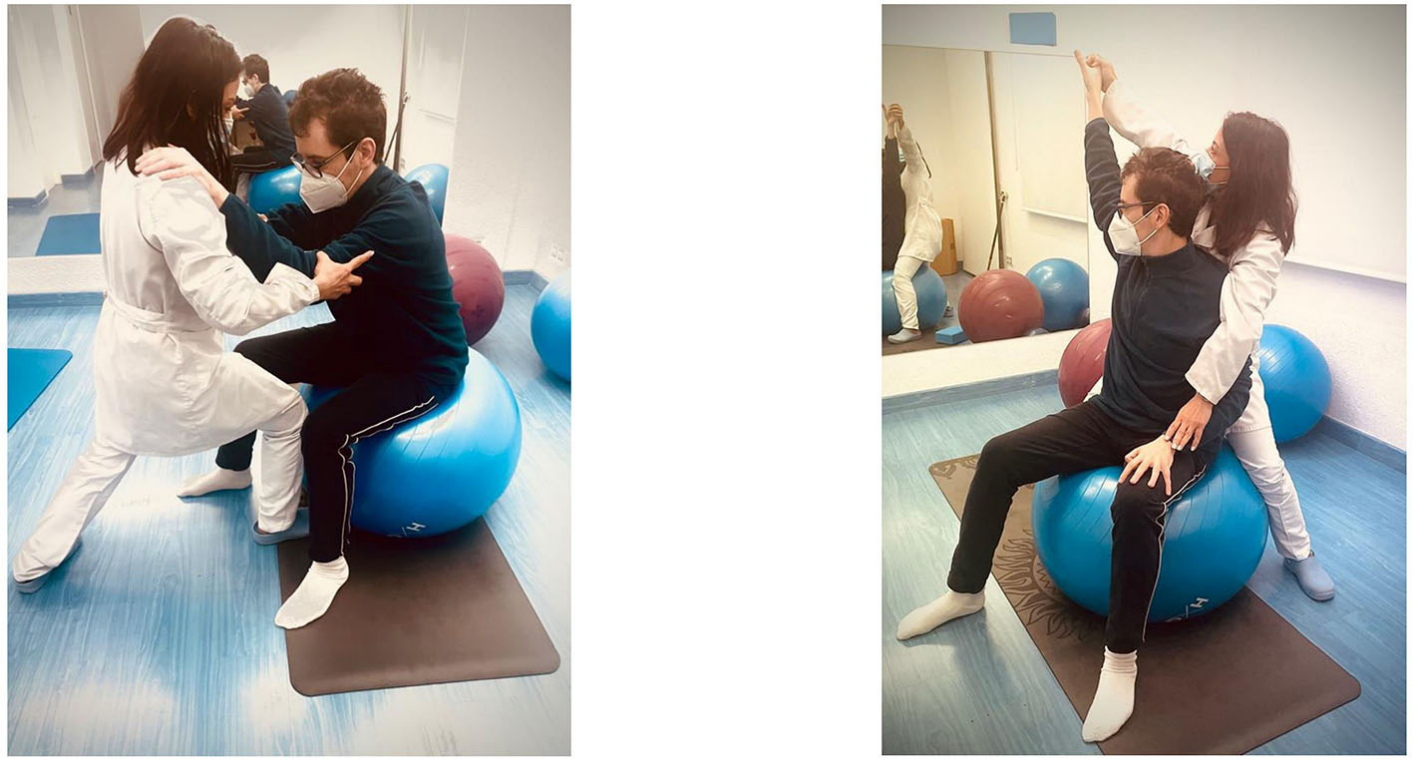
Neurorehabilitation treatment according to the Bobath therapy.

**What (procedures):** For the application of the Bobath concept, different postures will be applied to inhibit tone and abnormal movement patterns, facilitate normal movement, and stimulate muscular inactivity. To this end, pathological coordination patterns controlled by tonic activity (tonic reflexes) will be inhibited by controlling specific points. It will also facilitate normal coordination patterns controlled by straightening and balancing reactions. It will activate weak muscle groups in the trunk and in the lower and upper limbs to increase muscle tone. To do this, a precise stroke is performed with the therapist's outstretched fingers along the extension of the muscle or a number of muscles working in the same direction. Figure 2 shows the treatment according to the Bobath concept.

**Who will provide:** All interventions will be performed by a physiotherapist with more than 20 years of experience in treating people with neurological problems.

**How:** The physiotherapy technique will be performed individually twice a week for a duration of 45 min for a period of 12 months.

**Where:** All sessions will be conducted at the Faculty of Nursing and Physiotherapy, University of Salamanca (Spain).

**When and for how long:** Participants will receive two sessions per week for 12 months. The average duration of the session will be ~45 min. The same weekly schedule will always be respected for each of the participating subjects.

**Tailoring**: Due to the nature of the intervention and the wide heterogeneity of the subjects, the sessions will be individualized and tailored to each subject.

**How well (planned):** Therapy supervision will be provided through weekly meetings between therapists and investigators. In addition, a thorough control of the subjects' attendance at the sessions will be carried out.

**How well (actual):** Not applicable.

**Figure 3:**
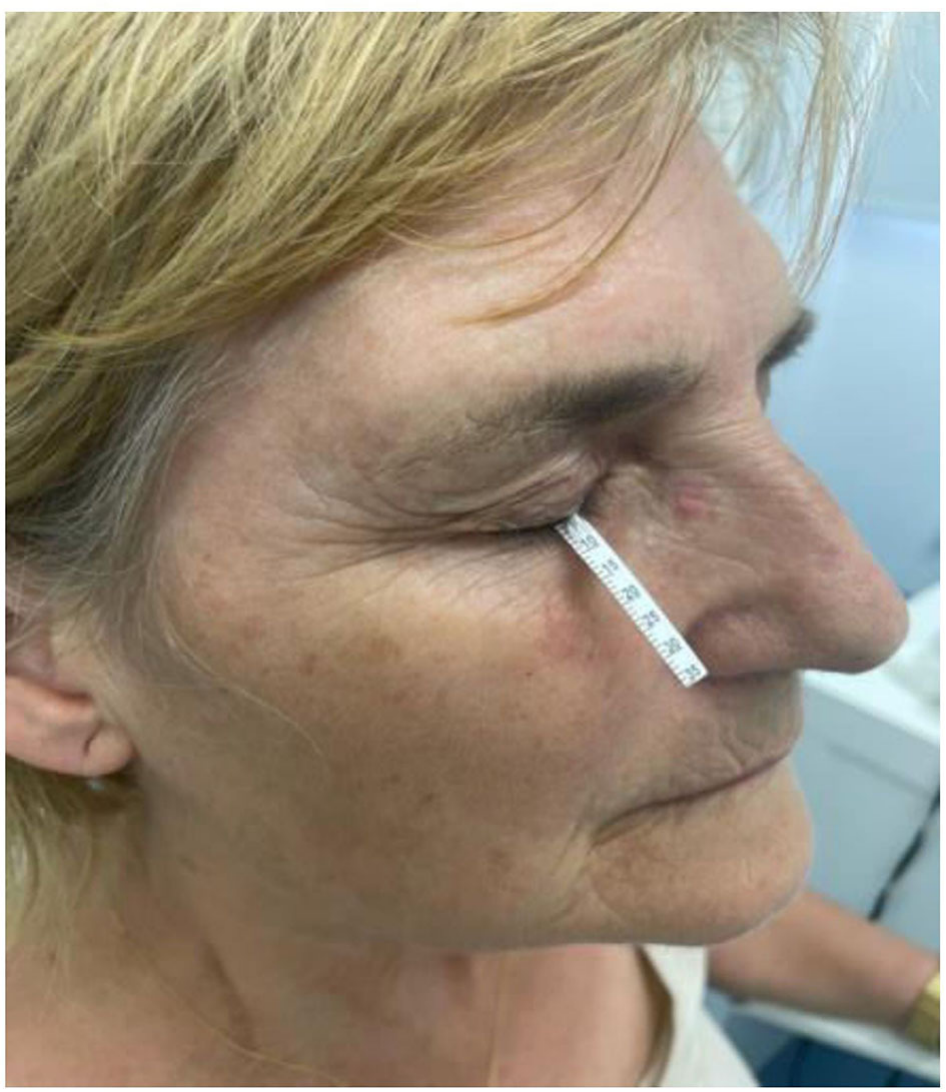
Schirmer's test uses paper strips placed in the lower eyelid, and its score is determined by the length of the moistened area of the strips (using the scale packaged with the strips). Tears are usually collected until the patient's eyelid normally moistens 20 mm of each paper strip.

**Extraction of samples:** Tear samples from MS patients will be collected at the Institute of Neurosciences of Castilla y León. For this, tears will be collected on graduated Schirmer's strips, as previously described by Pieragostino et al. (28). The Schirmer's test will be purchased from Praxisdienst GmbH & Co KG (Longuich, Germany), and the strips will be gently placed on the lower eyelid. Since the strips are graduated, they will be kept on the lower eyelid until the signal reaches the indicated mark. Subsequently, the strip will be placed in a 2.0-ml Eppendorf tube and stored at −80°C until further analysis. Figure 3 shows an example of tear extraction.

### Sample analysis

Lipids will be extracted from paper strips moistened with the patient's tears. To this end, a modified method of Bligh as described by Hijazi et al. (29) will be used. Briefly, the samples will be extracted with chloroform-methanol-water (2:1:1, in vol.) and mixed with corresponding internal standards (heptadecanoic acid for lipid analysis and protein calibration standard I (Bruker Daltonics) for proteomic analysis). After centrifugation at 2,000 × *g* for 15 min at 4°C, the resulting organic phase, the interphase, and the aqueous phase will be isolated.

The organic layer will be evaporated to dryness under a nitrogen stream. The final residue will be resuspended in a solution of methanol-chloroform (2:1, v/v) to a concentration higher than 5 pmol/ml and then analyzed directly in the chromatograph. The concentration of fatty acids in the sample will be determined in a Waters Acquity UPLC™ H-Class-Xevo TQS™ chromatograph coupled to a triple quadrupole mass spectrometer [LC-MS/MS(QqQ)], which allows performing nominal mass and is available at the General Mass Spectrometry Service (Nucleus) of the University of Salamanca. Different commercial fatty acid standards will be used to determine the retention time of each fatty acid.

The aqueous and interphase phases will be evaporated to dryness in a Speedvac and resuspended in 2.5% ACN and 50% TFA for protein detection using MALDI-TOF-TOF (matrix-assisted laser desorption/ionization time of light) AutoFlex mass spectrometry III of the Proteomics Unit of the Biomedical Research Institute of Neurosciences of Castilla y León. For automated data analysis, the obtained spectra will be processed using the MALDI Biotyper 3.0 program (Bruker Daltonics GMbH, Leipzig, Germany).

### Outcomes

In the baseline assessment, all variables, including sociodemographic variables, will be measured. Subsequently, all outcome variables will be measured at 3, 6, and 12 months and coded by a person external to the intervention.

Personal and sociodemographic variables are as follows:

- Age (years).
- Sex (male or female).
- Weight (kg), height (m), body mass index (BMI; kg/m^2^).
- Study level (no studies, basic education, secondary school, or superior studies).
- Marital status (single, married, separated, divorced, or widowed).
- Time since diagnosis of MS (years and months).
- Smoking (yes or no).
- Pharmacological treatment (name and number of pills per day).
- Comorbidities (name).

#### Primary outcome variables

**Figure 4:**
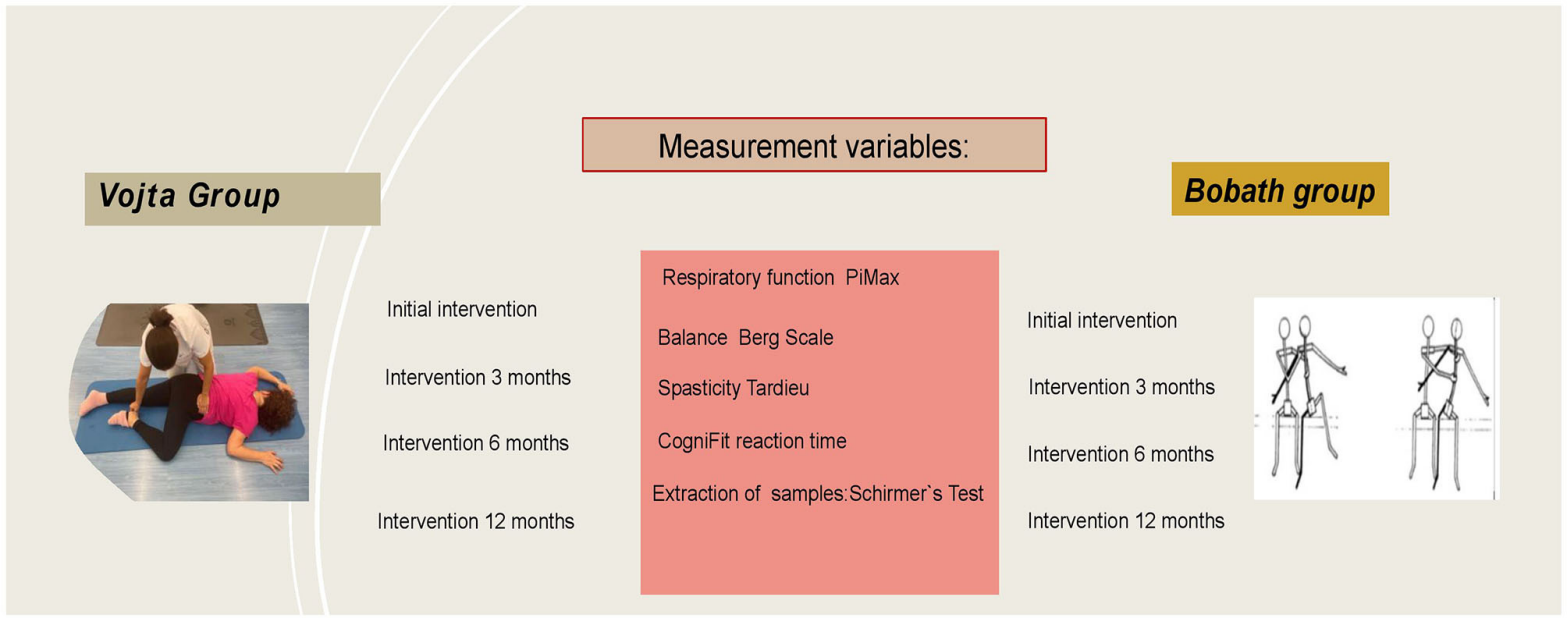
Timeline.

The primary outcome measures are respiratory function, balance, spasticity, reaction time, and quantification of lipid biomarkers. Figure 4 shows the timeline of the intervention and the evaluations of the different variables.

- **Respiratory function:** Maximum inspiratory pressure (PIMax) is the maximum pressure generated by the inspiratory muscles when performing a forced inspiration; in practice, it is a simple and global evaluation of the strength of the inspiratory musculature. Its knowledge is of great importance since it allows us to compare it with published reference values, use it in the programming of inspiratory training, and check its evolution over time, thus assessing the effectiveness of a respiratory rehabilitation program or inspiratory training (30). PiMax will be measured with the Baluue peak flow meter inspirometer.
- **Balance:** The Berg Balance Scale was developed in 1989 to measure balance in the elderly (31). The scale consists of 14 items, scored from 0 to 4, which are added to make a total score between 0 and 56; a higher score indicates better balance. The items vary in difficulty, from sitting in a chair to standing on one leg. The Berg Balance Scale takes ~10–15 min to complete. It requires a chair, a stopwatch, a ruler, and a step. Although the Berg Balance Scale was originally developed to measure balance in the elderly, it is now commonly used to measure balance in people with varying conditions and disabilities (32).
- **Spasticity:** The Tardieu scale has been suggested as a better instrument for classifying spasticity because it uses slow and fast speeds in accordance with the definition of spasticity and can differentiate between neural and biomechanical contributions to resistance to passive movement (33–35).
- **Reaction time**: Reaction time refers to the amount of time that elapses from when we perceive something until we respond accordingly. Therefore, it is the ability to detect, process, and respond to a stimulus. Any type of disorder that involves perception, information processing, or motor problems will affect reaction time. Therefore, response time is a cognitive ability that is very sensitive to alterations. Reaction time will be measured through the CogniFit program (36). CogniFit is a computerized cognitive training program that was found to be effective in improving reaction time and cognition (37, 38).
- **Lipid biomarkers**: Cicalini et al. (24) analyzed the lipid profile of 30 different phospholipids using lipidomics, resulting in a significantly lower concentration of sphingomyelin in subjects with MS. In this study, in addition to analyzing the amount of sphingomyelin, we will analyze phospholipids such as phosphatidylcholine, phosphatidylserine, phosphatidylethanolamine, and free fatty acids such as arachidonic acid, arachidic acid, and linoleic acid. The objective of analyzing these lipids plus sphingomyelin relies on their predominance in cell membranes and myelin sheaths, which are involved in MS pathology.

### Power and sample size

The sample size was calculated using the G^*^power Software (version 3.1.7), resulting in a minimum of 54 participants for the development of the study. The estimated effect size for the main outcome measures established in this study was 0.25. A power of the statistical test of 0.95, an alpha error of 0.05, a correlation between repeated measurements of 0.5 (two groups, two measurements), a sphericity correction coefficient of 1, and a loss percentage of 20% are taken into account.

### Allocation and randomization

Participants will be randomly assigned to one of the two intervention groups, namely, the reflex locomotion group or the Bobath therapy group.

Concealed allocation will be performed with a computer-generated random table of numbers created by an external statistician who will not be involved in the analysis or interpretation of the results. Individual, sequentially numbered tokens will be prepared for the random assignment. The cards will be placed in sealed, opaque envelopes, which will be opened by an external evaluator. The assignment to treatment will be revealed to the participants after the initial results are collected.

### Blinding

Participants will be blinded to their group assignment. Because of the nature of the intervention, the clinician cannot be blinded. In addition, the statistical analysis will be performed by an independent statistician who will be unaware of the intervention group. The person in charge of data collection will also be blinded.

### Statistical methods

Statistical analysis will be performed using the SPSS 27.0 statistical software for Windows (SPSS Inc., Chicago, IL; version 27.0). The normality of the sample will be checked using the Shapiro-Wilk test, and if it follows a normal distribution, an analysis of variance (ANOVA) of repeated measures *p* will be used, with time (pre-treatment and post-treatment) as an intra-group factor and Vojta and Bobath groups as the inter-group factor, all with a Bonferroni correction. Statistical analysis will be performed with a confidence level of 95%, so a *p*-value of < 0.05 will be considered significant values.

## Discussion

Different studies point to the effectiveness of reflex locomotion in improving balance in neurological patients and in diseases such as multiple sclerosis (39, 40).

There is evidence that reflex locomotion (Vojta) and the Bobath concept can improve the consequences of multiple sclerosis, such as alterations in balance, spasticity, respiratory function, or reaction time (5, 16, 39, 41). However, to date, previous studies have not attempted to biologically explain why this enhancement occurs.

This randomized clinical trial aims to demonstrate that the clinical improvement obtained by these patients may be due to an improvement in lipid markers, which are biomarkers of disease progression. Improving clinical parameters in patients with multiple sclerosis is a challenge in neurodegenerative diseases of the central nervous system (42).

The study of reflex locomotion (Vojta) and the Bobath concept could simultaneously improve the expression of lacrimal biomolecular markers that could be related to the myelination process and be co-related with parameters such as balance, spasticity, and reaction, and may influence similarly the pimax values of patients with multiple sclerosis (4, 43).

Analyzing the studies that precede this essay and that compare reflex locomotion and the Bobath concept without being related to myelination processes through the study of molecular markers present in the tear, should be a strong starting point for new lines of research.

### Potential impact and significance of the study

As previously described, MS is characterized as an inflammatory demyelinating disease of the CNS in which axonal and axonal loss demyelinating inflammatory disease of the CNS in which there is also axonal loss, and remyelination of the lesions is not always complete due to various factors and causes (44).

The possible existence of genetic and/or microenvironmental factors that could block oligodendrocyte differentiation and myelin production is the main oligodendrocyte differentiation and myelin production, which is the main focus of the search for its etiopathogenesis. But equally, the mechanisms of axonal activation and rehabilitative therapies to improve the quality of life of these patients continue to be a research line for a better interpretation of the clinical research, the clinic, and a correct approach (45).

There are few comparative studies on the effectiveness of reflex locomotion vs. the Bobath concept as rehabilitative therapies in people with MS, and none to date has analyzed in parallel the expression of lacrimal biomolecular markers that could participate in the processes of myelination, balance, respiratory parameters, spasticity, and reaction time. This is the reason why we highlight the relevance of our hypothesis, proposing a new methodology in the physiotherapeutic approach to the disease.

Given the special importance and relevance of scientific interest in research into the different factors and causes that may relate myelination processes to the development and progression of multiple sclerosis, the physiotherapeutic approach has always been aimed at carrying out programmes that produce changes in the postural control of these patients in the short term, either using reflex locomotion or the Bobath method.

Previously, a team led by Lopez et al. (39) performed interventions in two groups using balance scales such as the Berg Balance Scale and the Tandem tests, obtaining very positive results regarding better postural control in these patients.

Other authors, such as the Carratalá-Tejada et al. (46), beyond balance, have assessed fatigue using tools such as the Performance Oriented Mobility Assessment (POMA), the Fatigue Severity Scale (FSS), and the instrumental analysis of gait recorded using the Vicon Motion System^®^, managing not only to improve these spatio-temporal parameters but also significantly improving the increase in joint ROM, the length of the stride, and the double support in standing position.

The study of balance, fatigue, muscle strength, spasticity, and even neuroproprioceptive facilitation in these patients using the Bobath method by the group of researchers led by Pavlikova et al. (47) and Angelova et al. (48) supports the hypothesis of adaptive plasticity, and they consider it reasonable to focus on trunk training to achieve positive effects on the upper limb based on the analysis of the aforementioned scales.

However, no study to date refers to the simultaneous analysis of scales such as Pimax, balance, spasticity, reaction time, and lipid analysis from tear ducts using the Folch method, the reflex locomotion, and the Bobath method, or even establishes the comparison of these two methods (together and separately), using the analysis of molecular biomarkers and therefore relating them to myelination processes.

In this way, the study of our research is highlighted, which proposes a new approach to physiotherapy programs in patients with multiple sclerosis, which would delay the loss of physical capacity in these patients, enable the improvement of evolutionary processes during the disease, and therefore increase the quality of life.

### Limitations

#### Risk factor 1: selection and number of patients

A total of 50 patients diagnosed with RRMS will be selected. They will come from Salamanca's capital and its province. Considering that the MS Care Unit of Salamanca Hospital evaluates more than 300 patients diagnosed with MS disease in general, an initial screening will be carried out to select 50 individuals who could benefit from these therapies. Individuals belonging to different MS associations from nearby provinces to Salamanca (Salamanca) may also participate if there is a need to select additional participants in this assay.

#### Risk factor 2: delay in implementing the therapy within the initially scheduled period

Although different factors may delay the implementation of planned therapies, one of the most relevant could be the appearance of MS attacks in evaluated patients during the assay. This would probably lead to a delay in taking samples and carrying out the proposed therapy. To counteract this circumstance, a new subject in the study and/or an extension in project duration may be considered depending on the time risk.

#### Risk factor 3: inadequate sample collection

If samples are not collected at the scheduled time points, they may be delayed until the next session. It is not considered crucial to collect samples exactly at the same time.

#### Risk factor 4: inadequate sample processing

Sample processing must be carried out thoroughly to avoid the loss of irreparable data. For this reason, tears from both eyes will be collected to guarantee an extra sample if needed.

## Ethics and dissemination

The study is being conducted in accordance with the protocol and principles of the current version of the Declaration of Helsinki (49). Permission to conduct the study was granted by the Ethics Committee of the University of Salamanca on March 2023 (ref: 896).

All patients in the study will be informed about the objective of the study and will give their written informed consent to participate in the study. The patients' right to privacy will be respected, the applicable data protection laws in force will be observed, and the anonymity of all study participants will be guaranteed when data are presented in scientific journals. The medical data of the patients will be considered confidential, and disclosure to third parties will be forbidden. To ensure proper confidentiality, identification numbers will be randomly assigned to each participant. The physiotherapeutic treatments will be adapted to each patient, so the sessions will be individualized. The risk of therapy-related adverse events is minimal. To date, no adverse effects of these therapies have been reported in people with multiple sclerosis (5, 6, 39, 46).

However, if serious adverse events occur during or after the study, these will be monitored by the study team and the physicians as part of standard therapy. All serious adverse events will be documented and reported immediately (within a maximum of 24 h) to the principal investigator of the study.

## Ethics statement

The studies involving human participants were reviewed and approved by Ethics Committee of University of Salamanca (record number 896). The patients/participants will provide their written informed consent to participate in this study prior to their participation. Written informed consent was obtained from the individual(s) for the publication of any potentially identifiable images or data included in this article.

## Author contributions

JS-G, AA-C, and AV are the principal investigators of this study, refined the protocol, wrote the manuscript, and contributed to the design of the study. AC-G supervised the study. All authors have contributed equally to this manuscript, critically revised the protocol for important intellectual content, and approved the final manuscript.
